# Xpo7 is a broad-spectrum exportin and a nuclear import receptor

**DOI:** 10.1083/jcb.201712013

**Published:** 2018-07-02

**Authors:** Metin Aksu, Tino Pleiner, Samir Karaca, Christin Kappert, Heinz-Jürgen Dehne, Katharina Seibel, Henning Urlaub, Markus T. Bohnsack, Dirk Görlich

**Affiliations:** 1 Department of Cellular Logistics, Max Planck Institute for Biophysical Chemistry, Göttingen, Germany; 2 Bioanalytical Mass Spectrometry Group, Max Planck Institute for Biophysical Chemistry, Göttingen, Germany; 3 Institute for Clinical Chemistry, University Medical Center Göttingen, Göttingen, Germany; 4 Institute for Molecular Biology, University Medical Center Göttingen, Göttingen, Germany

## Abstract

Exportins bind cargo molecules in a RanGTP-dependent manner inside nuclei and transport them through nuclear pores to the cytoplasm. CRM1/Xpo1 is the best-characterized exportin because specific inhibitors such as leptomycin B allow straightforward cargo validations in vivo. The analysis of other exportins lagged far behind, foremost because no such inhibitors had been available for them. In this study, we explored the cargo spectrum of exportin 7/Xpo7 in depth and identified not only ∼200 potential export cargoes but also, surprisingly, ∼30 nuclear import substrates. Moreover, we developed anti-Xpo7 nanobodies that acutely block Xpo7 function when transfected into cultured cells. The inhibition is pathway specific, mislocalizes export cargoes of Xpo7 to the nucleus and import substrates to the cytoplasm, and allowed validation of numerous tested cargo candidates. This establishes Xpo7 as a broad-spectrum bidirectional transporter and paves the way for a much deeper analysis of exportin and importin function in the future.

## Abstract (precis)

Aksu et al. explore the vast cargo spectrum of exportin7/Xpo7 and present anti-Xpo7 nanobodies that acutely inhibit Xpo7’s transport cycles in living cells. Their expression selectively blocks nuclear enrichment of import cargoes as well as nuclear exclusion of export cargoes, establishing Xpo7 as a novel bidirectional nuclear transport receptor.

## Graphical Abstract

**Figure:**
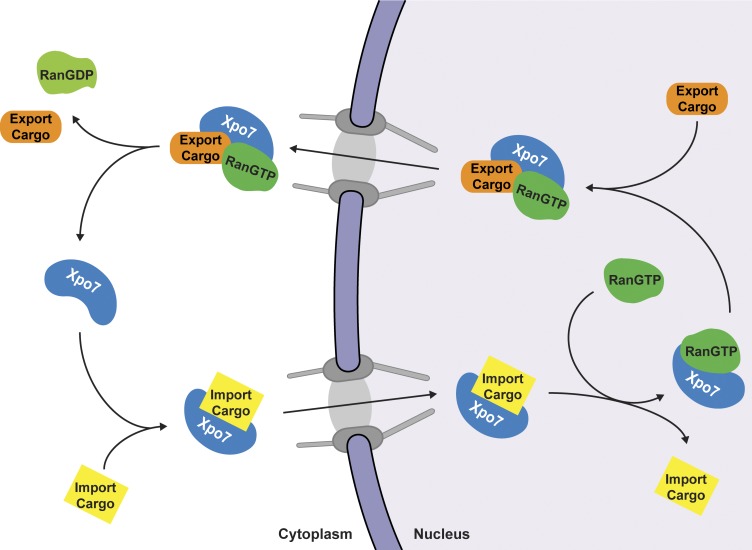

## Introduction

Correct partitioning of individual proteins, protein complexes, and ribonucleoprotein particles between cell nucleus and cytoplasm is essential for eukaryotic life. This requires a sophisticated barrier system as well as shuttling nuclear transport receptors (NTRs) that mediate translocation of cargoes either into or out of the nucleus. The barrier system retains cargoes at their destinations and suppresses an uncontrolled intermixing of nuclear and cytoplasmic contents. It comprises the two concentric nuclear membranes as well as the FG domain–guarded central channel of nuclear pore complexes (NPCs).

The members of the importin-β superfamily represent the largest NTR class. They draw energy from the RanGTPase system to control interaction with their cargoes in a compartment-specific manner (Görlich and Kutay, 1999). Importins bind substrates at low RanGTP levels in the cytoplasm, translocate through NPCs, release cargo upon encountering RanGTP in the nucleus, and finally return to the cytoplasm for GTP hydrolysis and binding of the next cargo. Exportins operate the other way around. They recruit cargoes together with RanGTP inside nuclei and release them into the cytoplasm.

Mammals use eight different exportins in parallel (Güttler and Görlich, 2011; Matsuura, 2016), and some of them are specialized in transport of just a single type of cargo. CAS/Xpo2, for example, exports only the nuclear import adapter importin-α (Kutay et al., 1997); for Xpo6, the only known function is to counteract leakage of actin into nuclei (Stüven et al., 2003). The other extreme is CRM1/Xpo1 (Fornerod et al., 1997; Fukuda et al., 1997; Ossareh-Nazari et al., 1997; Stade et al., 1997; Wolff et al., 1997), which exports a very large number of structurally unrelated cargoes, roughly 1,000 in human cells (Thakar et al., 2013; Kırlı et al., 2015). This is possible because CRM1 recognizes transplantable short linear motifs that reside within disordered protein regions. These motifs are called nuclear export signals (NESs) and comprise four to five critical hydrophobic Φ-residues that dock into five dedicated pockets of this exportin (Fischer et al., 1995; Wen et al., 1995; Dong et al., 2009; Monecke et al., 2009). Docking can occur in various backbone conformations and in different orientations, thus allowing CRM1 to accept NESs with variable Φ-spacings (Güttler et al., 2010; Fung et al., 2015). Several toxic compounds, such as leptomycin B, incapacitate CRM1 by modifying a cysteine at its NES-binding site and therefore block this pathway (Hamamoto et al., 1983; Nishi et al., 1994; Wolff et al., 1997; Dong et al., 2009; Monecke et al., 2009; Sun et al., 2013). This has been exploited extensively for discovering and validating CRM1 cargoes.

Finally, one yeast and two mammalian NTRs are known to function in both import and export. Yeast Msn5p exports phosphorylated Pho4p (Kaffman et al., 1998) and imports RPA (Yoshida and Blobel, 2001). Importin 13 mediates nuclear import of UBC9 and retrieves the translation initiation factor eIF1A back to the cytoplasm (Mingot et al., 2001; Bono et al., 2010). Xpo4 is an exporter for eIF5A and also imports transcription factors such as Sox2 (Lipowsky et al., 2000; Gontan et al., 2009; Aksu et al., 2016).

RanBP16/Xpo7 (Kutay et al., 2000; Mingot et al., 2004) has so far been one of the least-characterized NTRs. Several binding partners have been identified but only two export cargoes, namely RhoGAP1 and 14-3-3 σ, were actually shown to be exported by this exportin (Mingot et al., 2004). Yet Xpo7 seems to play a major role in erythropoiesis, where the B-isoform of Xpo7 is heavily induced. Its knockdown interferes with terminal erythroid differentiation and more specifically with nuclear condensation, loss of histones from nuclei, and ultimately the enucleation of the prospective red blood cells (Hattangadi et al., 2014). However, it has remained unclear whether this erythropoietic function relates to Xpo7-mediated nuclear export.

We revisited the transport function of Xpo7 and identified not only ∼200 potential export substrates but also, surprisingly, ∼30 import cargoes. For validation, we used newly developed, powerful tools, namely plasmid-encoded nanobodies that act as acute intracellular inhibitors of Xpo7 transport cycles in vivo without the need for a lengthy protein depletion or biochemical fractionation. These nanobodies can now be used for Xpo7 cargo discovery, cargo validation, and studying developmental roles of this bidirectional transporter in more complex systems.

## Results and discussion

### Identification of potential Xpo7 cargoes

**Figure 1.**
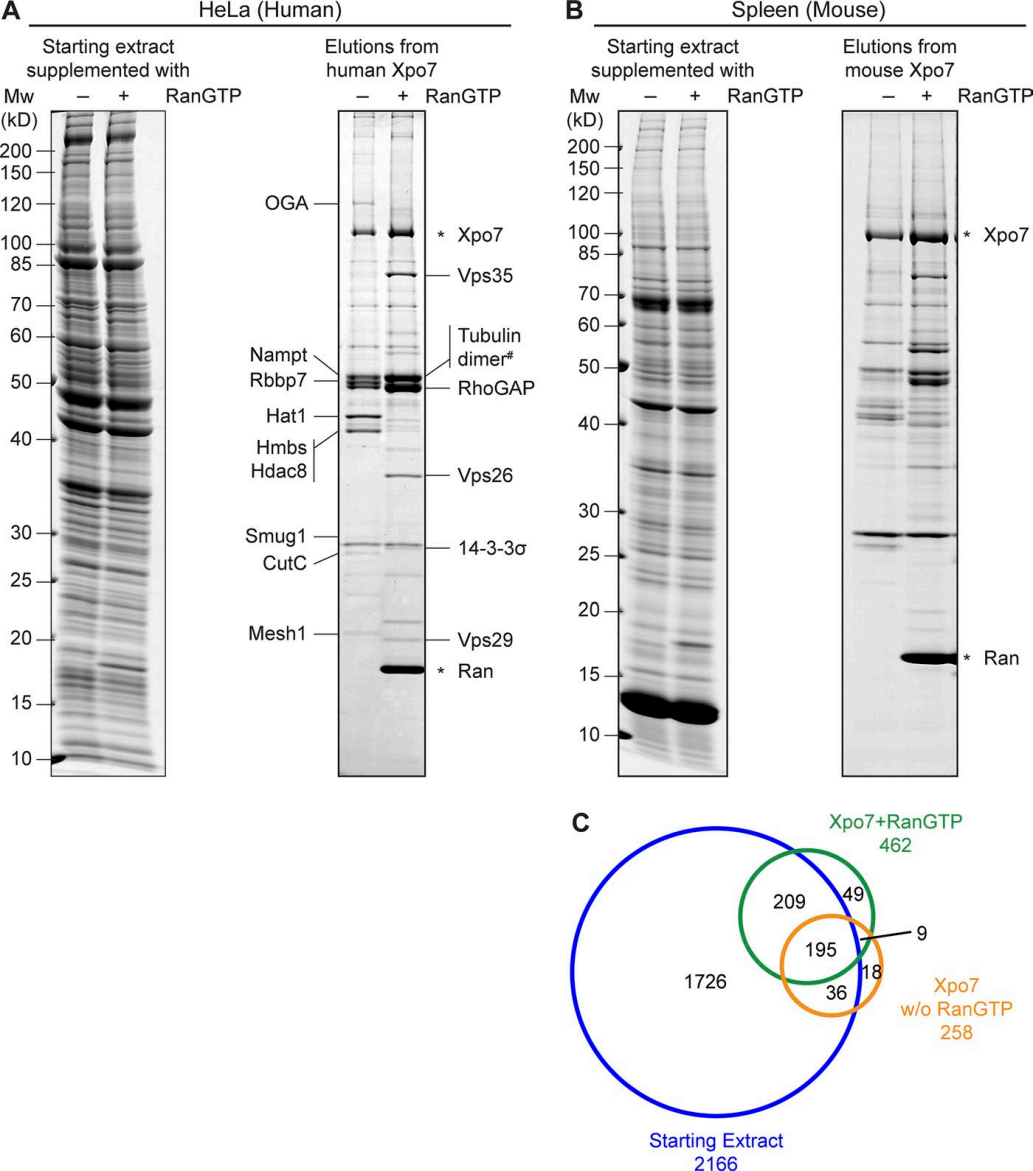
**Identification of novel Xpo7 binders. (A)** Xpo7 tagged with the ED domains of protein A was immobilized on anti–protein A beads and incubated with 2 ml hypotonic HeLa extract (Abmayr et al., 2006) in the absence or presence of 5 µM RanGTP (Q69L ΔC terminus mutant). 1/2,000 of the starting extracts and 1/10 of the bound fractions were analyzed by SDS-PAGE followed by Coomassie staining. Indicated bands were identified by MS. Tubulin dimer^#^ denotes tubulin α 4A and tubulin β 4B as the predominant forms. **(B)** Xpo7 affinity chromatography was performed as in A, but an extract from mouse spleen was used as starting material. Mw, molecular weight. **(C)** Starting extract (–Ran), Xpo7–RanGTP, and Xpo7 without Ran samples in B were analyzed by MS. The Venn diagram represents the number of identified unique proteins.

To obtain a comprehensive overview of Xpo7 transport cargoes, we used, in a first set of experiments, human HeLa cell extract as a starting material. One aliquot was supplemented with a GTP state-locked Ran (Q69L ΔC) mutant to mimic intranuclear binding conditions (Bischoff et al., 1994; Izaurralde et al., 1997), whereas another aliquot was left without RanGTP addition and served as a cytoplasmic sample. The two samples were then incubated with immobilized Xpo7, and bound proteins were eluted after thorough washing of the matrices and analyzed by SDS-PAGE and Coomassie staining (Fig. 1 A).

This revealed several prominent RanGTP-dependent Xpo7 binders, which included the two previously validated export cargoes RhoGAP1 and 14-3-3 σ (Mingot et al., 2004). In addition, α- and β-tubulin and the Vps35–Vps29–Vps26A complex (Bonifacino and Hurley, 2008) were identified as major bands in the sample containing RanGTP. A nuclear import activity of Xpo7 had not yet been demonstrated. Therefore, it was quite remarkable that the analysis also revealed several Ran-sensitive Xpo7 binders that represent potential import cargoes.

In a next step, we switched from the HeLa cancer cell line to an organ of extramedullary hematopoiesis, namely mouse spleen, which also showed the highest Xpo7 expression levels of all analyzed tissues (unpublished data). We prepared a spleen extract and proceeded with Xpo7 affinity chromatography as described above (Fig. 1, B and C). This time, however, we subjected the samples to a deep proteomic analysis by mass spectrometry (MS) and thereby identified 258 proteins bound to Xpo7 alone (representing potential import cargoes) and 462 proteins bound to the Xpo7−RanGTP complex (putative export cargoes). For comparison, 2,166 proteins were identified in the parallel analysis of the starting extract.

It is clear that not all identified proteins indeed represent true Xpo7 binders. In fact, any noninteractor from the starting extract could contaminate a bound fraction, and the probability of detection increases with a protein’s abundance in the starting material. To address this problem, we used the intensity-based absolute quantitation (iBAQ) strategy (Schwanhäusser et al., 2011) to quantify all detectable proteins in the input extract as well as in the bound materials. This allowed estimation of how strongly a given protein had been enriched within the Xpo7-bound and Xpo7–RanGTP-bound fractions. These enrichment factors can be seen as a measure of Xpo7’s affinity toward a certain cargo. The numbers also quantify the stimulating or antagonizing impact of RanGTP on a given Xpo7 interaction, and thus allow a grouping of interactors into potential export or import cargoes. Considering also the sensitivity of MS detection, we combined these numbers into import and export scores for all identified binders (see Materials and methods). Data S1 lists these scores along with the corresponding UniProt identifiers and information obtained by data mining such as functional descriptions or assembly of individual proteins into protein complexes.

The identified cargo candidates are functionally and structurally diverse. On the export side, they include the heterotrimeric Vps35–Vps29–Vps26A complex, which had the highest export score of 9.3–9.7 and whose three subunits were found in a roughly 1:1:1 stoichiometric ratio. The complex is part of the retromer and mediates membrane trafficking from endosomes to the TGN (Bonifacino and Hurley, 2008). Possibly, export by Xpo7 prevents such membrane trafficking from being initiated from the nuclear interior. Likewise, the ATPase Asna1/Trc40, which posttranslationally delivers tail-anchored proteins to the ER (Stefanovic and Hegde, 2007), behaves like an Xpo7 export substrate, perhaps to prevent inappropriate insertions into the inner nuclear membrane.

The tetratricopeptide repeat–containing protein Ttc39c, a protein implicated in ciliary function (Xu et al., 2015), was also identified with a very high export score (9.3). It later served as a positive control for cargo validation. Kinases contribute >10% of the high-scoring export candidates, examples being the choline/ethanolamine kinase (Gallego-Ortega et al., 2009); the receptor-interacting serine/threonine-protein kinase 1 (cell death protein RIP); MAP3K5, NEK7, and NEK9; the protein-tyrosine kinase 2-β; casein kinase II; and the eukaryotic elongation factor 2 kinase. Also, a few phosphatases have been identified, namely the protein phosphatase 1G, the protein phosphatase 6 complex, and the tartrate-resistant acid phosphatase type 5.

Remarkably, histone H4 showed up with a high export score of 6.7. This supports the view that the function of Xpo7 in erythropoiesis relates to histone export before enucleation (Hattangadi et al., 2014). We would, however, assume that H4 does not bind Xpo7 directly but rather through an as yet unidentified histone chaperone that acts as an export adapter. Not only should this chaperone be developmentally regulated, but one would also predict that only its histone-loaded form is recognized as an export cargo. Several histone-interacting proteins with high export scores were also identified. These include the histone-lysine *N*-methyltransferase SMYD3 (Hamamoto et al., 2004), the histone chaperone Anp32e (Obri et al., 2014), and the histone chaperone and phosphatase PP2A inhibitor protein SET (Adachi et al., 1994; Li et al., 1996; Beresford et al., 2001).

The potential import cargoes again include several histone-interacting proteins such as the Hat1 histone acetyltransferase (Makowski et al., 2001), its partner the histone-binding protein RBBP7/RBAP46 (Campos et al., 2010), and the histone deacetylase Hdac8 (Hu et al., 2000). We also found numerous enzymes such as the protein O–GlcNAcase (OGA/MGEA5; Gao et al., 2001), the nicotinamide phosphoribosyltransferase Nampt (Gallí et al., 2010), the single-strand selective monofunctional uracil DNA glycosylase Smug1 (a DNA repair enzyme; Haushalter et al., 1999), porphobilinogen deaminase/hydroxymethylbilane synthase (Hmbs), and the copper homeostasis protein CutC homologue (Li et al., 2005). Furthermore, several tRNA maturation enzymes and several large complexes, such as the THO complex, also behave like Xpo7 import cargoes.

### Biochemical validation of cargo–Xpo7 interactions

**Figure 2.**
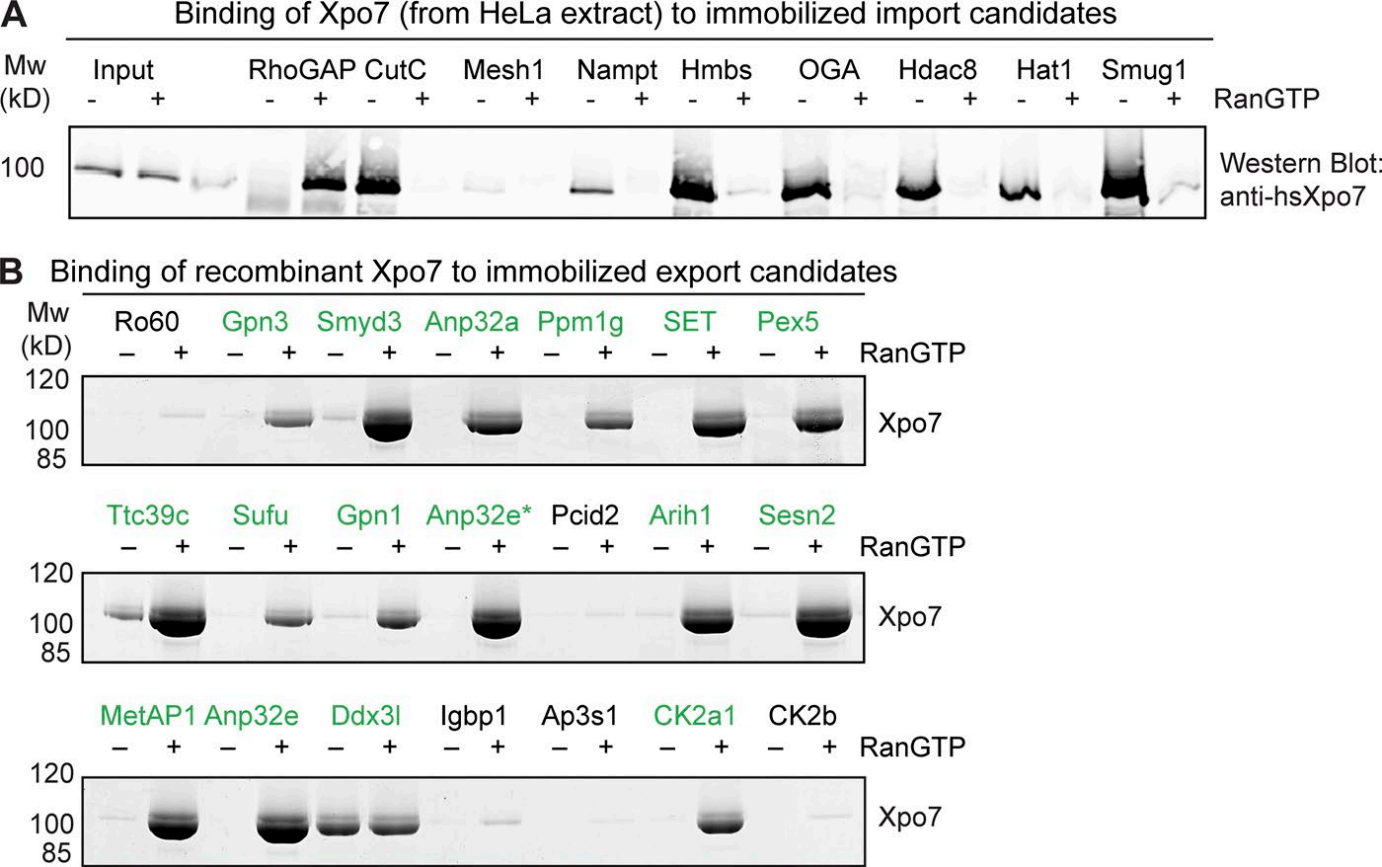
**Validation of cargo candidates as true Xpo7 binders. (A)** H14-ZZ-*bd*NEDD8–tagged cargo candidates (human proteins) were immobilized on anti–protein A beads and incubated with a cytoplasmic HeLa extract in the absence or presence of 3 µM RanGTP. After washing off unbound material, the immobilized candidates and bound proteins were eluted by *bd*NEDP1 protease cleavage. 1/250 of the starting extracts and 1/10 of the eluates were analyzed by SDS-PAGE followed by immunoblotting with an anti-hsXpo7 antibody recognizing a C-terminal epitope (Mingot et al., 2004). **(B)** Binding assays were performed as in A, but candidates (from mouse) were incubated with purified recombinant Xpo7 in the absence or presence of RanGTP, and the eluates were analyzed by SDS-PAGE and Coomassie staining. Anp32e* corresponds with Anp32e isoform 3 (E9Q5H9). Mw, molecular weight.

As a first step of validation, we recombinantly expressed several import cargo candidates, immobilized them to Sepharose, and incubated them with a HeLa cell extract as a source of Xpo7 (Fig. 2 A). After extensive washing, bound fractions were eluted and immunoblotted against Xpo7. This revealed a possibly false-positive hit (Mesh1, identified so far only in HeLa pulldowns). CutC, Nampt, Hmbs, the protein O–GlcNAcase, Hdac8, Hat1, and Smug1, however, were clearly positive and recruited Xpo7 in a RanGTP-sensitive manner.

By performing binding experiments in an all-recombinant system, we also confirmed the direct and RanGTP-dependent interaction of Xpo7 with 15 potential export cargoes (Fig. 2 B). These included both subunits of the Gpn1–Gpn3 GTPase complex, which appears to function in the assembly and nuclear import of the RNA polymerase II complex (Carré and Shiekhattar, 2011), the histone chaperones Anp32a and 32e (acidic leucine-rich nuclear phosphoprotein), the protein phosphatase Ppm1g, protein SET, the peroxisomal import receptor Pex5, and the casein kinase II isoform α 1 (catalytic subunit). Some of the tested export candidates showed only very weak binding such as the regulatory casein kinase II β subunit, whose recruitment to Xpo7 apparently occurs largely through the α subunit.

### Xpo7 can mediate nuclear export of tubulin

**Figure 3.**
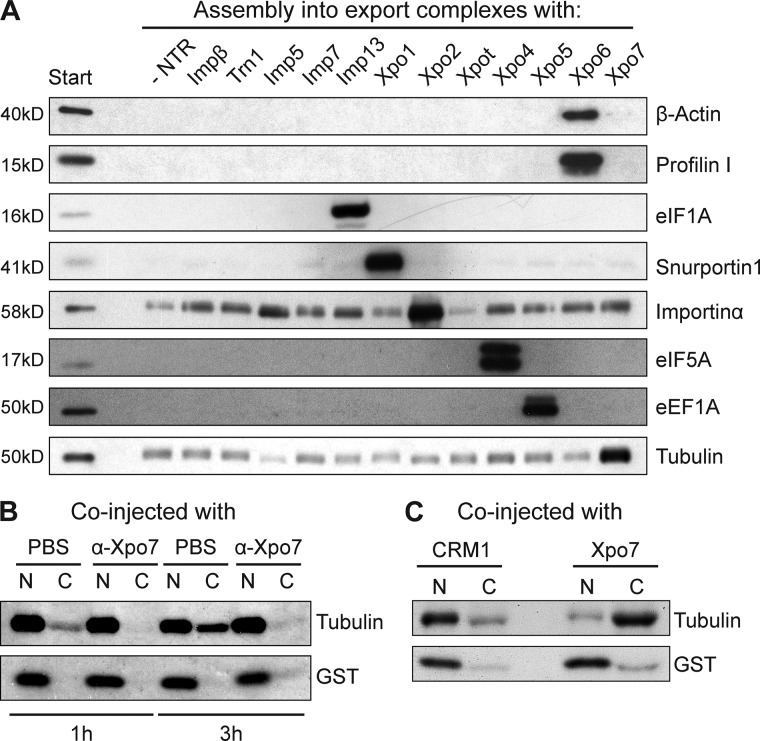
**Xpo7 binds to and mediates nuclear export of tubulin. (A)** A cytoplasmic HeLa extract was depleted of endogenous NTRs by the phenyl-Sepharose method (Ribbeck and Görlich, 2002). The sample was split, and single importins or exportins were added to the extract for formation of export complexes with ZZ-tagged RanGTP immobilized on IgG-Sepharose. Bound fractions were analyzed by immunoblotting with a monoclonal antibody recognizing α-tubulin. The panel also shows immunoblots against known cargoes of NTRs other than Xpo7. The bound fractions each correspond with 20× the amount of loaded starting extract. Note that tubulin was enriched only in the sample containing Xpo7. The signal observed with other NTRs resembles background binding to the matrix as seen for the sample without addition of any transport receptor. **(B)** [^35^S]-labeled GST (nuclear injection marker) and human tubulin α6 were injected into the nuclei of *Xenopus* oocytes. Anti-Xpo7 antibodies (6 mg/ml) or 1× PBS were coinjected. Oocytes were dissected 1 or 3 h postinjection, and the nucleocytoplasmic distribution of the labeled proteins was analyzed by SDS-PAGE followed by autoradiography. The antibodies blocked tubulin export. **(C)** Radiolabeled tubulin and GST were coinjected with human Xpo7 or CRM1/Xpo1 into the nuclei of *Xenopus* oocytes. 4 h postinjection, oocytes were analyzed as in B. Note that export of tubulin is strongly stimulated by coinjection of Xpo7.

The most abundant protein in the Xpo7–RanGTP-bound fraction was tubulin, probably binding as an α/β-tubulin dimer (Fig. 1 A). For validation, we performed a binding assay to a panel of NTRs that had been immobilized through RanGTP. This confirmed the preferential interaction of tubulin with Xpo7 (Fig. 3 A). Tubulin is known to be excluded from interphase nuclei of animal cells, perhaps to avoid untimely interactions with the numerous nuclear components that drive spindle formation in mitosis. Given that tubulin forms a dimer of ∼100 kD and further polymerizes, it is expected to leak into nuclei only rather slowly. Yet this does not rule out an active export from cell nuclei.

To look into this issue, we produced [^35^S]-labeled tubulin by in vitro translation in a reticulocyte lysate, injected it into nuclei of *Xenopus laevis* oocytes, and measured its efflux by analyzing manually dissected nuclear and cytoplasmic fractions 1 and 3 h later (Fig. 3 B). This revealed a slow nuclear exit of tubulin, which became essentially blocked by a coinjected anti-Xpo7 antibody. Coinjection of Xpo7, in turn, boosted the export strongly (Fig. 3 C), suggesting that tubulin is indeed an export cargo for Xpo7 and that this exportin is limiting for the rate of export.

### Nanobody-based export cargo validation

Microinjections into oocytes are a rather laborious way of testing the transport requirements of cargoes. We therefore aimed at a validation method that allows a higher throughput and is compatible with transfection assays in which GFP-tagged cargo candidates are transiently expressed in cultured cells. To this end, we considered camelid-derived single-domain antibodies, called nanobodies (Muyldermans, 2013), as inhibitors of Xpo7 transport cycles.

To obtain such nanobodies, we immunized an alpaca with recombinant human Xpo7, isolated lymphocytes after the last boost, prepared an immune library, and retrieved specific binders by phage display selections on the immobilized antigen. This way, we obtained several different classes of anti-Xpo7 nanobodies. Two of them (D11 and D18) function also under reducing conditions, bind Xpo7 with high affinity, and are able to specifically isolate Xpo7 in a total HeLa cell extract (Fig. S1). Indeed, MS analysis revealed that only Xpo7 itself and some Xpo7 interactors (such as CutC) copurified with these nanobodies, confirming their specificity.

The actual validation relies on cotransfection of two plasmids. One plasmid confers transient expression of GFP-fused cargo candidates. The other plasmid expresses a nanobody as well as an NES-RFP fusion that not only serves as a control for transfection but also tests whether nuclear exclusion by CRM1/Xpo1 remains intact.

**Figure 4.**
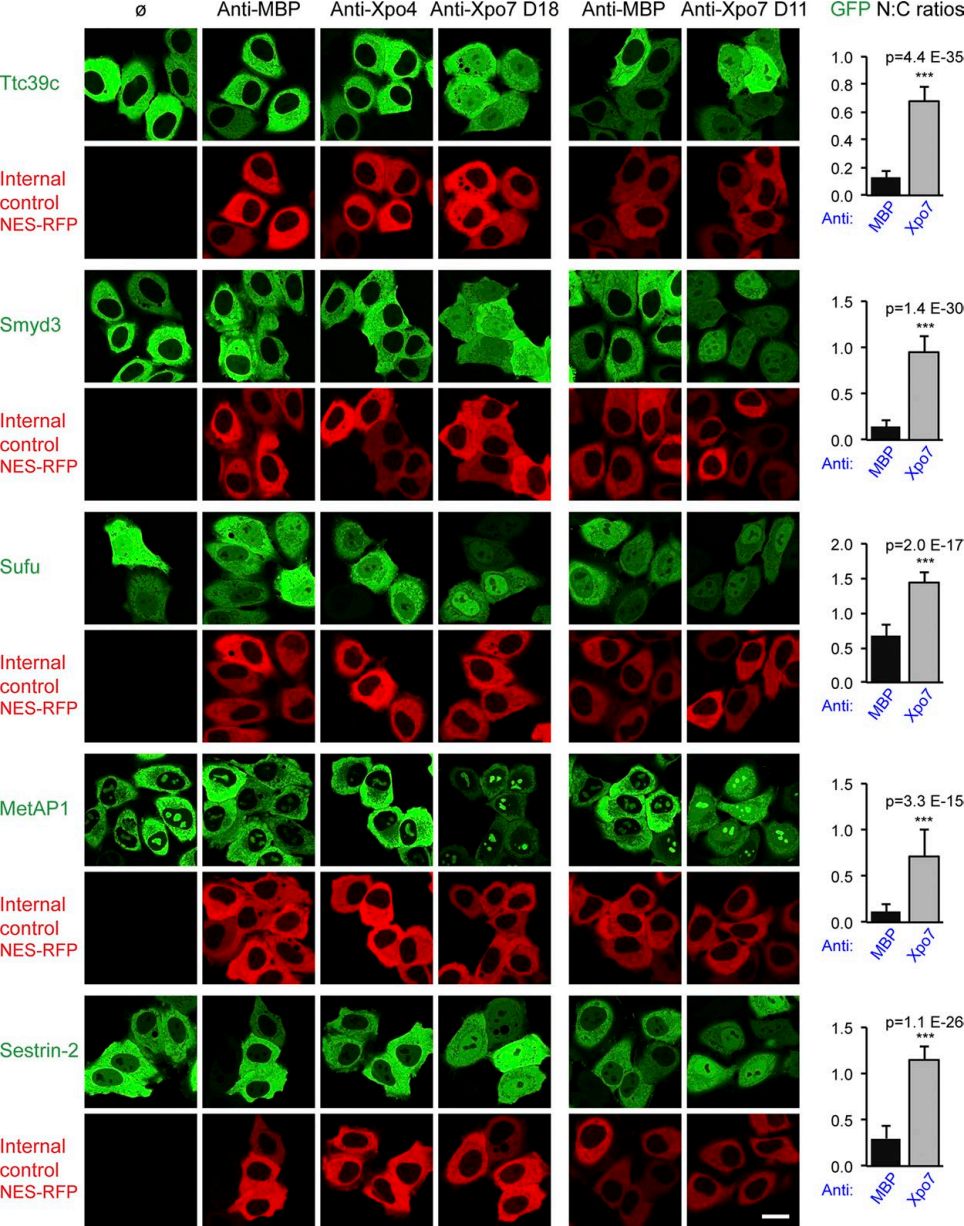
**Validation of export cargo candidates.** GFP-fused candidate proteins from mouse were transiently expressed in HeLa cells, and their subcellular localization was recorded in live cells by confocal fluorescence microscopy. The effect of the nanobodies recognizing MBP, Xpo4, or Xpo7 (D18) was monitored by cotransfection with a vector encoding NES-RFP to stain the cytoplasmic compartment. In a separate experiment, anti-Xpo7 nanobody D11 was tested. Bar, 20 µm. The predominant cytoplasmic localization of Ttc39c (Q8VE09), Smyd3 (Q9CWR2), MetAP1 (Q8BP48), and Sestrin-2 (P58043) was disrupted only by anti-Xpo7 nanobodies, which led to nuclear accumulation. This suggests that these proteins can leak into nuclei and that they are kept cytoplasmic at steady state by Xpo7-dependent export. A milder disruption was observed for Sufu (Q9Z0P7), where block of Xpo7 increased the relative nuclear GFP signal. UniProt entry names are listed in parentheses. On the right, mean ratios of nuclear/cytoplasmic (N:C) GFP concentrations are plotted, with anti-MBP and anti-Xpo7 nanobody results averaged (*n* = 20–40). Error bars represent SD. Statistical significance (***, p < 10^−6^) was assessed using an unpaired two-sided *t* test. Means ± SD of each sample are listed in Table S1.

The repeat protein Ttc39c was tested first because it had a high Xpo7 export score (+9.3), it binds Xpo7 directly in a RanGTP-stimulated manner (Fig. 2 B), and its GFP fusion is strictly excluded from nuclei of undisturbed cells (Fig. 4). It was striking to see that the anti-Xpo7 nanobody D18 indeed abolished this nuclear exclusion and led to an equilibration of Ttc39c between nucleus and cytoplasm (Fig. 4). Thus, the protein can enter nuclei but is retrieved back to the cytoplasm by Xpo7, and the Xpo7-specific nanobody can inhibit this export process.

Several controls confirmed the specificity of the effect: (a) another anti-Xpo7 nanobody with unrelated CDRs (D11) caused the same Ttc39c relocation to the nucleus (Fig. 4, right column); (b) the Ttc39c localization did not change when either an anti–maltose-binding protein (MBP; *Escherichia coli* MalE) or an anti-Xpo4 nanobody was coexpressed; and (c) the anti-Xpo7 nanobodies relocated neither the previously identified Xpo1 cargo MetAP2 (Fig. S2 A) nor the cotransfected NES-RFP fusion reporter. Thus, the nanobodies D11 and D18 are specific for the Xpo7 export pathway and do not cross-inhibit the parallel export by Xpo1.

In a next step, we extended the analysis to additional export cargo candidates. We observed that the histone lysine *N*-methyltransferase Smyd3 (Hamamoto et al., 2004) is also excluded from nuclei by Xpo7. The enzyme should exert its function inside nuclei, and we therefore consider Xpo7 as a negative regulator. Furthermore, Xpo7 depletes the methionine amino peptidase MetAP1 from nuclei, which binds to 80S ribosomes and cotranslationally cleaves the starting methionine from numerous nascent polypeptides (Vetro and Chang, 2002; Giglione et al., 2015). Note that the second enzyme of this pathway MetAP2 is excluded by Xpo1 (Kırlı et al., 2015). The leucine sensor and TORC1 activator Sestrin-2 (Saxton et al., 2016) also shows nuclear exclusion that is clearly Xpo7 dependent.

A slightly weaker effect was evident for Sufu (suppressor of fused), which is regarded as a negative regulator of the hedgehog signaling pathway (Svärd et al., 2006). Sufu interacts directly with Xpo7 (Fig. 2 B); however, as part of a regulatory network, it forms complexes with several other proteins. It remains to be seen which of those complexes are actually subject to Xpo7-mediated export.

We also encountered proteins that showed a clear RanGTP-stimulated Xpo7 interaction in pulldown assays and yet did not shift into nuclei upon Xpo7 inhibition in HeLa cells (Fig. S2 B). For Pex5, this is plausible because it is also a cargo of Xpo1 (Kırlı et al., 2015), and this appears sufficient to maintain a cytoplasmic localization. In the case of Gpn1, we assume two reasons: first, the complex appears also to be a cargo of CRM1 (Kırlı et al., 2015), and second, it is likely that the Gpn1–Gpn3 complex constitutes the actual export species and that endogenous Gpn3 levels are too low to capture all of the transiently expressed GFP-Gpn1 fusion. In the future, further research is needed to explore how the nucleotide-bound states of the two GTPases impact their interaction with Xpo7.

### Validation of Xpo7 import cargoes

CutC, a homotetramer and one of the strongest Xpo7 interactors (import score, 9.7), turned out to be a predominantly nuclear protein. Anti-Xpo7 nanobodies changed this localization only slightly (Fig. S2 C and Table S1). This could imply that Xpo7 has no import activity. A more likely explanation, however, is that CutC uses several nuclear import pathways and that a loss of Xpo7 can thus be compensated for.

**Figure 5.**
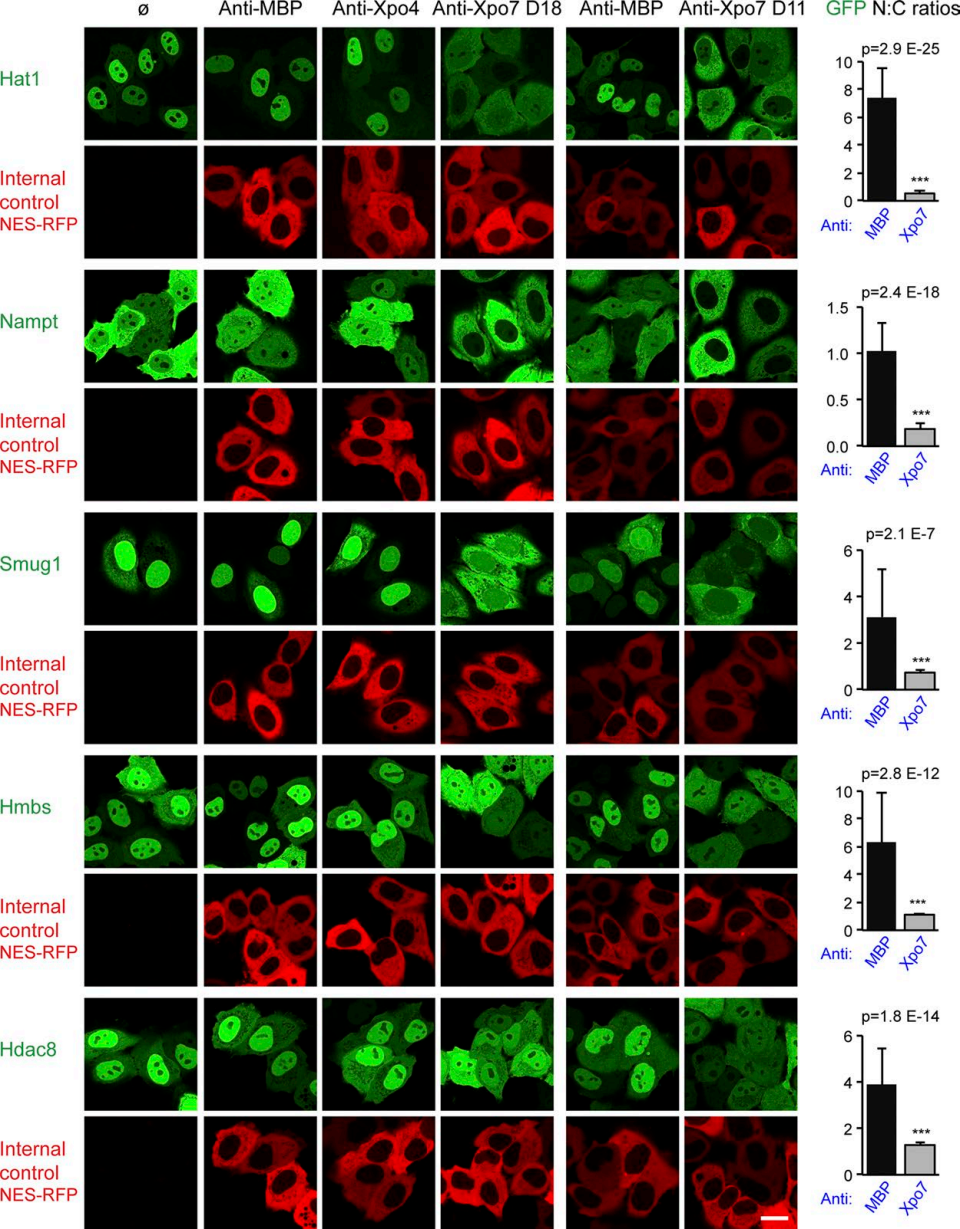
**Validation of import cargo candidates.** The analysis of potential import cargo candidates was performed as described in Fig. 4 and included the human proteins Hat1 (O14929), Nampt (P43490), Smug1 (Q53HV7), Hmbs (P08397), and Hdac8 (Q9BY41). Note that the anti-Xpo7 nanobodies shift the here-validated import cargoes to a more cytoplasmic localization. Error bars represent SD. ***, p < 10^−6^. Bar, 20 µm.

Hat1, Nampt, Smug1, and Hmbs turned out, however, to be clear import substrates of Xpo7 (Fig. 5). Upon Xpo7 inhibition, their localization changed either from nuclear/predominantly nuclear to equilibrated/nuclear exclusion (Hat1, Smug, and Hmbs) or from an even nucleocytoplasmic distribution to nuclear exclusion (Nampt).

So far, we assessed the effects of Xpo7 inhibition with transiently overexpressed GFP fusions of the respective cargo candidates. To test the effect on an endogenous cargo, we next detected Hat1 in HeLa cells with the help of anti-Hat1 antibodies. Strikingly, its nuclear localization was lost in cells that expressed the D11, the D18, or a combination of both anti-Xpo7 nanobodies (Fig. S3). Collectively, these results establish that Xpo7 functions not only as a broad-spectrum exportin, but also as a nuclear importer. It is thus, like importin 13 and Xpo4, a bidirectional transporter.

### Outlook

A straightforward validation of cargoes and a direct visualization of transport activity have so far been the bottlenecks of functional Xpo7 studies. This can now be bypassed by using the inhibitory anti-Xpo7 nanobodies described in this study. They interrupt Xpo7 transport cycles and thus relocate true Xpo7 cargoes. The system is as straightforward to use as leptomycin B for testing CRM1-dependent localization and is applicable to any transfectable or transducible cell type. Nanobody binding to Xpo7 results in an acute block of protein function as soon as the target has been titrated out. In contrast to siRNA knockdowns, the observed phenotype is also independent of the half-life of the targeted protein. Moreover, this should largely avoid compensatory mechanisms that are likely to occur in survivors of genetic perturbations.

We plan to expand this system in several directions. First, we would like to cover all importins and exportins and thereby come closer to the ultimate goal of a comprehensive functional description of all nuclear transport pathways. An additional anti-Xpo4 nanobody (E07) is already described in this study (Figs. 4, 5, and S4). It served as a control to prevent Xpo7 cargoes from being mislocalized by inhibition of a parallel pathway. We also show, however, that E07 prevents nuclear exclusion of Xpo4’s main export substrate, namely hypusinated eIF5A (Fig. S4). Nanobody E07 is thus a suitable tool to analyze Xpo4-dependent transport.

Several nanobodies can be combined in transfection/transduction experiments, allowing the intricate issue of redundancy between individual transport routes to be addressed. Another obvious direction is to dissect developmental processes. Indeed, it is tempting to establish transgenic mice that can express such inhibitory nanobodies in a (Tet-) inducible manner. In the case of Xpo7, this would allow the study of, e.g., its role in erythropoiesis and the effects of an acute loss of function in the most relevant biological context of a live animal.

## Materials and methods

### Xpo7 affinity chromatography

Spleen extract preparation was performed in 20 mM Hepes/KOH, pH 7.5, 50 mM KOAc, 2 mM MgOAc, and 250 mM sucrose in the presence of protease inhibitors (10 µM E-64, 10 µM aprotinin, 1 µM bestatin, 1 µM pepstatin A, and 50 µM chymostatin and leupeptin) and with the help of a SilentCrusher (Heidolph Instruments). The homogenate was cleared by centrifugation (4,500 *g* for 35 min at 4°C), and the supernatant was frozen and stored at −80°C. HeLa S100 extract (Abmayr et al., 2006) was supplied by the group of R. Lührmann (Max Planck Institute for Biophysical Chemistry, Göttingen, Germany), supplemented with 50 mM KOAc and 2 mM MgOAc, and stored at −80°C.

Thawed extracts were supplemented with 5 mM DTT and 50 mM KOAc and cleared by 1-h centrifugation (20,000 *g* at 4°C). Supernatants were first incubated with benzonase (30 U/ml), then with phenyl-Sepharose (low substitution to deplete endogenous NTRs) and precleared with anti–protein A beads (a tandem fusion of ZpA963; Lindborg et al., 2013). The flowthroughs were supplemented with 5 µM RanGTP (hsRanQ69L^5–180^; if applicable) and 2.5 µM cytochalasin B and then centrifuged in an AT4 rotor for 20 min at 4°C and 60,000 rpm.

2-ml extracts were incubated with 500 pmol preimmobilized mmXpo7A tagged with the ED domains of protein A. After 3 h at 4°C, the unbound material was removed, and the beads were washed thoroughly with 20 mM Hepes/KOH, pH 7.5, 110 mM KOAc, 5 mM MgOAc, 5 mM DTT, and 250 mM sucrose. Eluted proteins were analyzed by SDS-PAGE, Coomassie staining and tryptic digests, and MS.

### Sample preparation and instrumentation for MS

Separation of proteins was done by SDS-PAGE (4–12% Bis/Tris gradient gel; NuPAGE; Novex) and visualized by colloidal Coomassie staining. Proteins in excised gel pieces were digested as described previously (Shevchenko et al., 2006), with minor modifications. In brief, proteins were reduced with 10 mM DTT for 30 min at 55°C and alkylated with 55 mM iodoacetamide for 20 min at 25°C in the dark, both in 100 mM ammonium bicarbonate (ABC) buffer. Proteolysis was performed overnight at 37°C using trypsin (0.01 µg/µl final concentration; Serva) in 50 mM ABC buffer in presence of 5 mM CaCl_2_. Peptides were retrieved from the gel pieces by a series of extraction steps with 5% (vol/vol) formic acid, 50% (vol/vol) acetonitrile, and undiluted acetonitrile. Peptide-containing pooled fractions were concentrated by vacuum evaporation in a SpeedVac. Dried peptides were dissolved in 20 µl sample solvent (2% acetonitrile and 0.05% trifluoracetic acid for mouse spleen samples; 5% acetonitrile and 1% formic acid for HeLa samples; vol/vol). 8-µl fractions were analyzed by liquid chromatography–tandem MS (LC-MS/MS). Quantitative data from mouse spleen were acquired with a QExactive HF (Thermo Fisher Scientific). An LTQ Orbitrap XL (Thermo Fisher Scientific) was used for identifying Xpo7 binders from HeLa cells.

### LC-MS/MS QExactive HF analysis

In nano-LC, two sequential columns are used for desalting and chromatographic separation of extracted peptides. Both columns were packed in-house with spherical silica (ReproSil C18 AQ 120A; Dr. Maisch GmbH) with particle sizes of 5 µm in the precolumn (0.015 × 20 mm) and 3 µm in the tandem coupled analytical column (0.075 × 300 mm). Peptides were eluted using a 73-min linear gradient (5–44% acetonitrile in 0.05% TFA at a flow rate of 300 nl/min) on a Dionex Ultimate 3000 HPLC (Thermo Fisher Scientific) in-line coupled to a QExactive HF hybrid quadrupole-Orbitrap mass spectrometer (Thermo Fisher Scientific). The instrument was operated in data-dependent acquisition mode with a survey scan range of 350–1,600 m/z, a resolution of 60,000 at m/z 200, and an automatic gain control target value of 1 × 10^6^. Up to 30 of the most intense precursor ions with charge states ranging from 2 to 5 were selected at an isolation width of 1.6 m/z for higher collision-induced dissociation with a normalized collision energy of 28%. MS/MS scans were detected in the Orbitrap at a resolution of 15,000. Dynamic exclusion was set to 20 s.

### LC-MS/MS LTQ Orbitrap XL

Samples were injected onto a nano-LC system (1100 series; Agilent Technologies) consisting of a trapping column (0.015 × 20 mm) in-line with an analytical column (0.075 × 120 mm). Both columns were packed in-house with spherical silica material (5 µm; ReproSil C18 AQ 120; Dr. Maisch GmbH). Peptides were eluted and separated on the analytical column with a gradient of 3–37% buffer B (95% acetonitrile and 0.1% formic acid in H_2_O; vol/vol), an elution time of 90 min, and a flow rate of 300 nl/min. Online electrospray ionization MS was performed with a LTQ-Orbitrap XL (Thermo Fisher Scientific) operated in data-dependent acquisition mode using a TOP8 method. MS scans were recorded in the Orbitrap at the m/z range of 350–1,600 and a resolution of 30,000 at m/z 400. The eight most intense ions with charge states 2 and 3 were selected at an isolation width of 2.5 m/z for MS/MS. Fragmentation was generated by collision-induced dissociation activation (normalized collision energy, 37.5%). Fragment ions were acquired in the linear ion trap, and dynamic exclusion was set at 15 s with repeat count of 1.

### MS data analysis and annotation

MS raw files were processed with the MaxQuant software package (v.1.5.0.30), and measured spectra were searched against human and mouse FASTA sequence databases with the built-in Andromeda search engine (Cox and Mann, 2008; Cox et al., 2011). Common contaminants (e.g., keratins or serum albumin) were included in these databases, and reversed sequences of all entries were used for false discovery rate (FDR) estimations. The false discovery rate was set to 1% for proteins and modified and unmodified peptides. The Andromeda search engine parameters were as follows: carbamidomethylation of cysteine (fixed modification), oxidation of methionine and N-terminal protein acetylation (variable modifications), tryptic specificity with proline restriction including peptides with up to two missed cleavages, and minimum peptide length set to seven amino acids. Mass accuracy was set to 6 ppm in the MS survey scan, and MS/MS mass tolerance was set to 20 ppm.

For data annotation, protein complex lists from Ruepp et al. (2010) and Havugimana et al. (2012) together with the UniProt database (UniProt Consortium, 2015) were used to create functional groups. Further annotations were based on the UniProt database, from which relevant data were fetched and condensed to “simplified localization” (nucleus, cytoplasm, or both) and “flags” (transmembrane, ER, mitochondrial, and NPC proteins). The comprehensive and quantitative mouse spleen dataset has been deposited with the Pride Proteomics Identifications (PRIDE) database under accession no. PXD008746.

### Data processing for cargo identification

The molar fraction of a given protein within a sample was calculated by dividing the corresponding iBAQ intensity by the sum of iBAQ intensities of all proteins detected in that sample. Exportin and importin scores were calculated according to the following formulas:

$$\begin{array}{l} \mathrm{Export}\;\mathrm{score}=3*\mathrm{Log}\left({\mathrm{iBAQ}}^{\mathrm{withRan}}+\mathrm{DL}\right)-2*\\ \mathrm{Log}\left({\mathrm{iBAQ}}^{\mathrm{withoutRan}}+\mathrm{DL}\right)-\mathrm{Log}\left({\mathrm{iBAQ}}^{\mathrm{input}}+\mathrm{DL}\right); \end{array}$$

$$\begin{array}{l} \mathrm{Import}\;\mathrm{score}=3*\mathrm{Log}\left({\mathrm{iBAQ}}^{\mathrm{withoutRan}}+\mathrm{DL}\right)-2*\\ \mathrm{Log}\left({\mathrm{iBAQ}}^{\mathrm{withRan}}+\mathrm{DL}\right)-\mathrm{Log}\left({\mathrm{iBAQ}}^{\mathrm{input}}+\mathrm{DL}\right) \end{array}$$

where *DL* is the detection limit (100,000, median of iBAQ intensities of the least 30 abundant proteins). These formulas take into account (a) to what extent a protein is enriched from the input sample and (b) to what extent RanGTP influences that protein’s binding to Xpo7, thereby giving more weight to RanGTP stimulation/antagonism. The summand *DL* prevents infinite log values, for hits not detected in either input or one of the bound fractions. For such nondetected proteins, we thus assume that their abundance in a sample corresponds with the detection limit of the MS analysis. This yields conservative score estimates in cases of uncertainty.

We set a scoring threshold of 0.5 for a hit to be considered as import or export candidate. Hits with lower scores were removed from the list. This gave rise to 251 potential export and 41 potential import cargoes. The few hits that have positive values for both importin and exportin scores should be considered as RanGTP-independent Xpo7 binders. These proteins are listed in Data S1. Additionally, the interaction list has been submitted to the IMEx consortium (http://www.imexconsortium.org) through IntAct (Orchard et al., 2014) and assigned the identifier IM-26378.

### Protein expression and purification

All recombinant protein expression was in *E. coli*. Mouse or human Xpo7A was expressed as His-tagged proteins (also in combination with other tags) and purified either by Ni (II) chelate chromatography, imidazole elution, and gel filtration or by binding to Ni(II) beads, elution by a tag-cleaving protease (Frey and Görlich, 2014), and gel filtration. Candidate cargoes were expressed with an N-terminal His_10_-ZZ-*bd*NEDD8 tag and purified by Ni(II) chelate chromatography and imidazole elution. Expression and purification of His-tagged importins and exportins, ZZ-tagged RanQ69L used in Fig. 3 A, and tag-free RanQ69LΔC used in Fig. 2 were as described previously (Stüven et al., 2003; Aksu et al., 2016).

### Export assays in *Xenopus* oocytes

Export assays were essentially performed as described previously (Mingot et al., 2004). In brief, human tubulin α6 and GST were each cloned into an in vitro translation vector containing a T7 promoter and the 5′ UTR from *Xenopus* β-globin upstream of the ORFs. Midi preps of the resulting plasmids (Table 1) were then used to program 50-µl translation reactions in the T7 TNT reticulocyte system (Promega) supplemented with 1.67 mCi/ml [^35^S]methionine. Translations were performed for 90 min at 30°C. To remove free label, samples were diluted with 250 µl ice-cold 1× PBS and concentrated to 15 µl in a Nanostep centrifugal device with a 10-kD cutoff (Pall Corporation).

**Table 1.** Newly constructed plasmids used in the study

Identifier	Construct	Used in
pMA028	H10-ZZ-bdNEDD8-hsXpo7	Fig. 2
pKG031	H14-ZZ-bdSUMO-Ran5-180(Q69L)	Figs. 1 and 2
pMA018	H10-ZZ-bdNEDD8-hsRhoGAP	Fig. 2
pMA023	H10-ZZ-bdNEDD8-hsCutC	Fig. 2
pMA030	H10-ZZ-bdNEDD8-hsHAT1	Fig. 2
pMA031	H10-ZZ-bdNEDD8-hsMESH1	Fig. 2
pMA032	H10-ZZ-bdNEDD8-hsNAMPT	Fig. 2
pMA037	H10-ZZ-bdNEDD8-hsSMUG1	Fig. 2
pMA038	H10-ZZ-bdNEDD8-hsHMBS	Fig. 2
pMA039	H10-ZZ-bdNEDD8-hsMGEA5	Fig. 2
pMA040	H10-ZZ-bdNEDD8-hsHDAC8	Fig. 2
pMA241	H14-ZZ-bdNEDD8-mmTtc39c	Fig. 2
pMA244	H14-ZZ-bdNEDD8-mmTrove2	Fig. 2
pMA245	H14-ZZ-bdNEDD8-mmGpn3	Fig. 2
pMA248	H14-ZZ-bdNEDD8-mmSmyd3	Fig. 2
pMA250	H14-ZZ-bdNEDD8-mmSufu	Fig. 2
pMA251	H14-ZZ-bdNEDD8-mmGpn1	Fig. 2
pMA252	H14-ZZ-bdNEDD8-mmMetap1	Fig. 2
pMA253	H14-ZZ-bdNEDD8-mmAnp32e	Fig. 2
pMA253_v3	H14-ZZ-bdNEDD8-mmAnp32e_v3	Fig. 2
pMA255	H14-ZZ-bdNEDD8-mmPcid2	Fig. 2
pMA257	H14-ZZ-bdNEDD8-mmAnp32a	Fig. 2
pMA258	H14-ZZ-bdNEDD8-mmArih1	Fig. 2
pMA259	H14-ZZ-bdNEDD8-mmPpm1g	Fig. 2
pMA260	H14-ZZ-bdNEDD8-mmSet	Fig. 2
pMA262	H14-ZZ-bdNEDD8-mmSestrin-2	Fig. 2
pMA264	H14-ZZ-bdNEDD8-mmD1Pas1	Fig. 2
pMA268	H14-ZZ-bdNEDD8-mmIgbp1	Fig. 2
pMA269	H14-ZZ-bdNEDD8-mmPex5	Fig. 2
pMA271	H14-ZZ-bdNEDD8-mmAp3s1	Fig. 2
pMA274	H14-ZZ-bdNEDD8-mmCsnk2a1	Fig. 2
pMA275	H14-ZZ-bdNEDD8-mmCsnk2b	Fig. 2
pMA289	GFP-mmTtc39c	Fig. 4
pMA291	GFP-mmSmyd3	Fig. 4
pMA292	GFP-mmSufu	Fig. 4
pMA294	GFP-mmMetap1	Fig. 4
pMA300	GFP-mmSestrin-2	Fig. 4
pMA306	GFP-hsHAT1	Fig. 5
pMA307	GFP-hsNampt	Fig. 5
pMA309	GFP-hsSmug1	Fig. 5
pMA310	GFP-hsHmbs	Fig. 5
pMA312	GFP-hsHdac8	Fig. 5
pKoKeu145	GFP-MetAP2	Fig. S2
pMA293	GFP-mmGpn1	Fig. S2
pMA302	GFP-mmPex5	Fig. S2
pMA305	GFP-hsCutC	Fig. S2
pMA308	GFP-hsRbbp7	Fig. S2
pDG2298	H14-bdSUMO-Avi-hsXpo7	Immunization
pMA067	H14-bdNEDD8-mmXpo4	Immunization
pMA318	H14-Avi-Spacer(Sp)-SUMOStar-Sp-mmXpo4	Phage display
pMA319	H14-Avi-Sp-SUMOStar-Sp-hsXpo7	Phage display
pTP815	H14-Avi-Sp-bdNEDD8-Sp-MBP	Phage display
pMA389	Anti-Xpo4 Nb E07_IRES_NES-mRFP	Figs. 4, 5, and S4
pMA390	Anti-Xpo7 Nb D11_IRES_NES-mRFP	Figs. 4, 5, S2, and S3
pMA397	Anti-MBP-Nb TP250_IRES_NES-mRFP	Figs. 4, 5, S2, S3, and S4
pTP1225	Anti-Xpo7 Nb D18_IRES_NES-mRFP	Figs. 4, 5, S2, and S3
pTP1224	eEF1A promotor-GFP-eIF5A-P2A-DHS-P2A-DOHH	Fig. S4
pDG2968	T7 β-globin 5′UTR tubulin α6	Fig. 3
pDG2969	T7 β-globin 5′UTR GST	Fig. 3

Collagenase-treated *Xenopus* oocytes were prepared and used for nuclear injections with 15 nl sample volume as described previously (Jarmolowski et al., 1994). After indicated time points, oocytes were manually dissected, and proteins of the nuclear and cytoplasmic fractions were precipitated and analyzed by SDS-PAGE and autoradiography. The inhibitory antibody against a C-terminal epitope of *Xenopus* Xpo7 has been described previously (Mingot et al., 2004).

### Alpaca immunization

One female alpaca kept at the Max Planck Institute for Biophysical Chemistry was immunized four times at 3-wk intervals with 2 mg purified recombinant human Xpo7. For each injection, 500 µl of the purified protein in physiological buffer (20 mM Tris/HCl, pH 7.5, 150 mM NaCl, and 250 mM sorbitol) was mixed with 70 µl of a mild squalene/α-tocopherol/Tween-80 (22:24:10)–based adjuvant to form an oil-in-water emulsion.

### Selection of anti-Xpo7 nanobodies

The generation of nanobody immune libraries and the selection of antigen-specific nanobodies have been described previously (Pleiner et al., 2015, 2018) An initial set of nanobodies was obtained after two rounds of phage display selections with lower stringency that yielded a great variety of positive nanobodies. In a subsequent selection, the antigen concentration was lowered to 100 pM, yielding a new high-affinity class of nanobodies from which anti-Xpo7 nanobody D18 was derived. Nanobodies against Xpo4 and MBP (*E. coli* MalE) were obtained in the same way but using the respective target proteins as antigens for immunizations and baits in phage display.

### Native purification of Xpo7 from cytoplasmic HeLa cell lysate using nanobodies

HeLa S100 extract was adjusted to contain a final concentration of 150 mM NaCl, 2 mM MgCl_2_, and 5 mM DTT. After centrifugation in a TH-660 rotor for 1 h at 4°C at 48,000 rpm, the lipid-free supernatant was supplemented with 5 mM ATP and 5 µg/ml cytochalasin B. Biotinylated anti-Xpo7 nanobodies carried an N-terminal His_14_-biotin acceptor peptide (Avi-tag: GLNDIFEAQKIEWHE)-(GlySer)_9_-scSUMOStar-(GlySer)_9_-tag) and were prepared as described previously (Pleiner et al., 2015). They were then immobilized on magnetic Dynabeads MyOne streptavidin T1 (Thermo Fisher Scientific), and after washing with wash buffer (50 mM Tris/HCl, pH 7.5, 150 mM NaCl, 2 mM MgOAc, 2 mM DTT, and 0.005% [wt/vol] digitonin), the remaining biotin-binding sites on streptavidin were blocked by incubation with 50 µM biotin-PEG-COOH (from a 10-mM stock in dimethylformamide; Iris Biotech) in wash buffer. The blocked beads were then incubated with HeLa cell lysate for 1 h at 4°C. After two washes with wash buffer and two further washes with 50 mM Tris/HCl, pH 7.5, 500 mM NaCl, and 0.05% (vol/vol) Tween-20, bound proteins were eluted by cleavage with 500 nM SUMOStar protease (Liu et al., 2008) in 50 mM Tris/HCl, pH 7.5, and 300 mM NaCl for 30 min at 4°C. Eluted fractions were analyzed by SDS-PAGE followed by Coomassie staining and MS protein identification.

### Binding assays for cargo validation

0.75 µM ZZ-*bd*NEDD8–tagged candidates were incubated with 1 µM Xpo7 in the presence or absence of 2 µM RanGTP in a 20 mM Tris/HCl, pH 7.7, 100 mM NaCl, 2 mM MgOAc, and 2 mM DTT buffer. After 3-h incubation at 4°C, complexes were retrieved via tagged candidates by anti–protein A beads, eluted by *bd*NEDP1 protease cleavage, and analyzed by SDS-PAGE followed by Coomassie staining.

### Transient protein expressions in HeLa cells and fluorescence microscopy

Cargo candidates were cloned behind a GFP module in a modified pEGFP-C1 (Takara Bio Inc.) vector (eEF1A promoter). An NES (PKI)-RFP fusion was expressed from a control vector (CMV promoter; a modified version of pIRES puro 3; Takara Bio Inc.) in which nanobodies are cloned upstream of the NES-RFP ORF separated by a synthetic intron and an internal ribosomal entry site. HeLa Kyoto cells were grown in eight-well µ-slides (Ibidi) and transiently cotransfected using PolyJet transfection reagent (SignaGen) according to the manufacturer’s instructions. After 24 h, live cells were imaged in Leibovitz’s l-15 medium (supplemented with 10% FBS) by sequential scans with excitations at 488 and 561 nm using an SP5 confocal laser scanning microscope (Leica Microsystems) equipped with a 63× oil-immersion objective.

To detect endogenous Hat1, a recombinant rabbit monoclonal anti-Hat1 antibody (EPR18775; ab194296; Abcam) was used. After transfection, cells were fixed for 10 min with 4% (wt/vol) PFA in 1× PBS, PFA was quenched with 50 mM ammonium chloride, and cells were permeabilized with 0.3% (vol/vol) Triton X-100 and blocked with 5% (wt/vol) BSA before incubation with a 1:200 dilution of the primary antibody. The latter was detected with Alexa Fluor 488–labeled secondary anti–rabbit IgG nanobodies (Pleiner et al., 2018).

### Statistical methods

GFP or immunofluorescence signal intensities were quantified in ImageJ (National Institutes of Health). Means, SDs, and statistical significance were calculated in Excel.

### Online supplemental material

 Fig. S1 shows sequences of the newly raised nanobodies against MBP, Xpo4, and Xpo7; it also documents the specific interaction of the D11 and D18 nanobodies with Xpo7. Fig. S2 shows GFP fusions that do not change localization in response to anti-Xpo7 nanobodies. Fig. S3 demonstrates that anti Xpo7 nanobodies impede nuclear import of endogenous (untagged) Hat1. Fig. S4 documents that an anti-Xpo4 nanobody diminishes nuclear exclusion of eIF5A. Table S1 quantifies the impact of anti-Xpo7 nanobodies on the nucleocytoplasmic distribution of tested cargo molecules. Data S1 contains quantitative interaction data of ∼300 potential (import and export) cargoes with Xpo7.

## Supplementary Material

Supplemental Materials (PDF)

Data S1 (Excel)
