# Supplementary material for: Xpo7 is a broad-spectrum exportin and a nuclear import receptor
Source: J Cell Biol. 2018 Jul 2;217(7):2329–40. doi: 10.1083/jcb.201712013 (PMC6028547; doi:10.1083/jcb.201712013)
Supplement: Supplemental Materials (PDF) [file JCB_201712013_sm.pdf]

Supplemental material

Aksu et al., https://doi.org/10.1083/jcb.201712013

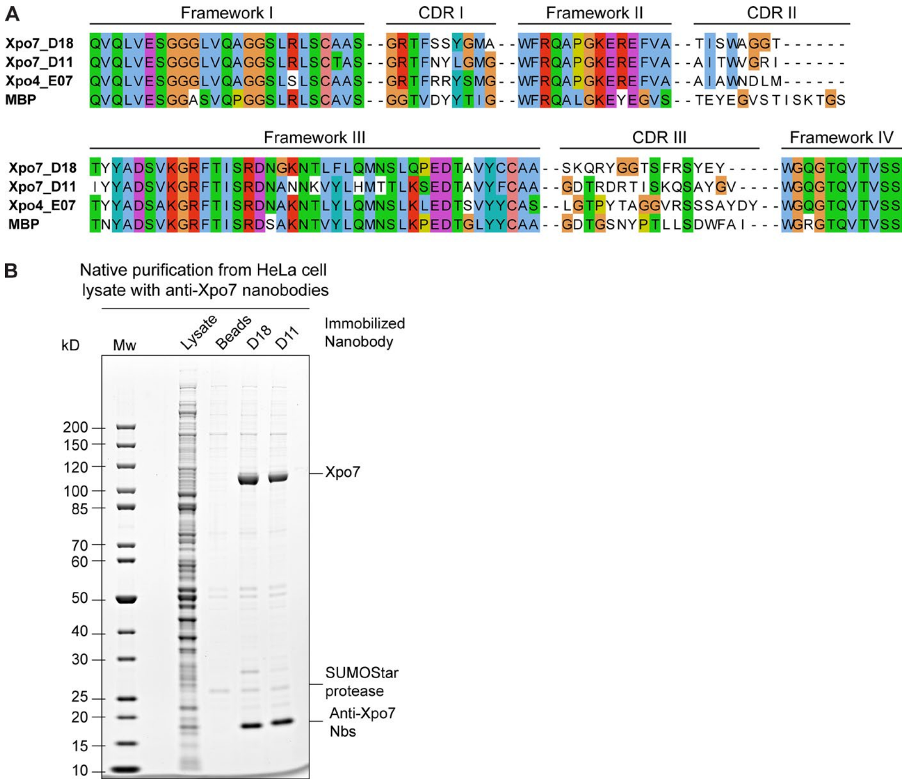

Figure S1. **Identification and specificity of anti-Xpo7 nanobodies. (A)** Sequence alignment of the nanobodies (Nbs) described in this study. **(B)** Biotinylated anti-Xpo7 nanobodies (Avi-SUMOstar tagged) were immobilized on streptavidin agarose and incubated with a HeLa extract. Beads were washed, and immobilized nanobodies along with the bound endogenous Xpo7 were released by SUMOstar protease cleavage. The starting lysate and eluates were analyzed by SDS-PAGE and Coomassie staining. An empty bead sample served as a negative control. Mw, molecular weight.

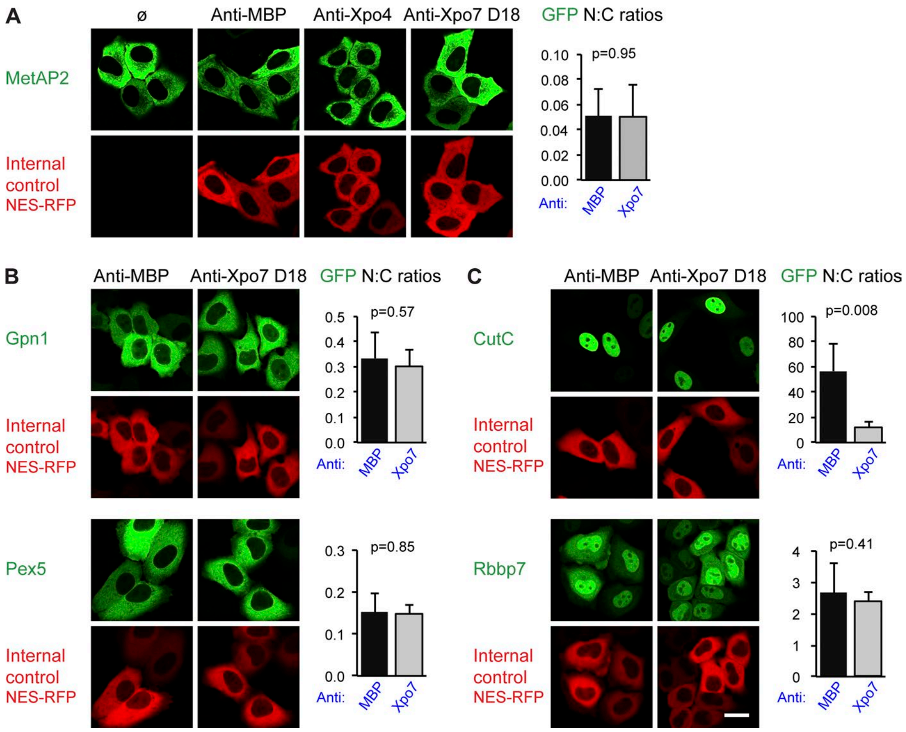

Figure S2. **A block of Xpo7 does not affect CRM1Xpo1 cargoes. (A)** As a negative control, MetAP2 (P50579), an export cargo of Xpo1/CRM1, was tested in transfection experiments as described in Fig. 4. The exclusive cytoplasmic localization of MetAP2 was not affected by any of the tested nanobodies. **(B)** Tested Xpo7 binders, whose intracellular distribution was not affected by anti-Xpo7 nanobody D18: Gpn1 (Q8VCE2) and Pex5 (O09012). Both are also CRM1 interactors (Kırlı et al., 2015), and Gpn1 probably forms a heterodimer with Gpn3. **(C)** CutC (Q9NTM9) and histone-binding protein Rbbp7 (Q16576) are potential Xpo7 import cargoes whose nuclear localization was only weakly impaired by anti-Xpo7 nanobody D18. CutC seems to use several redundant nuclear import pathways. In the case of Rbb7, we assume that its interaction partner Hat1 represents the actual Xpo7 import substrate. N:C, nuclear/cytoplasmic. Bar, 20 µm.

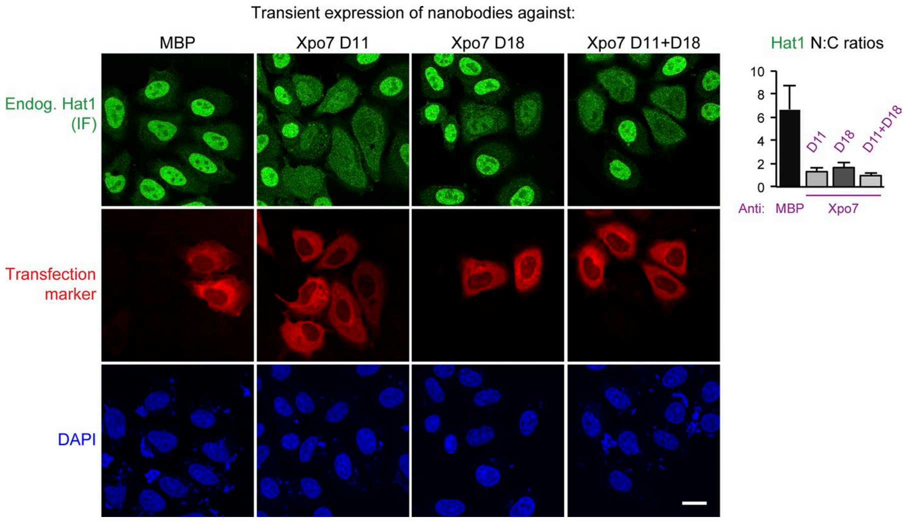

Figure S3. **Nuclear import of endogenous Hat1 is also blocked by anti-Xpo7 nanobodies.** Anti-MBP, Xpo7 D11, and Xpo7 D18 nanobodies were transiently expressed in HeLa cells, and their effect on Hat1 distribution was monitored by immunofluorescence (IF). Fixed cells were stained with an anti-Hat1 antibody and detected with Alexa Fluor 488–labeled secondary anti-rabbit IgG nanobodies (Pleiner et al., 2018). Untransfected cells served as negative controls. Although an anti-MBP nanobody had no effect, the anti-Xpo7 D11 and D18 nanobodies (or their combination) impaired Hat1 import and caused cytoplasmic accumulation. N:C, nuclear/cytoplasmic. Bar, 20 µm.

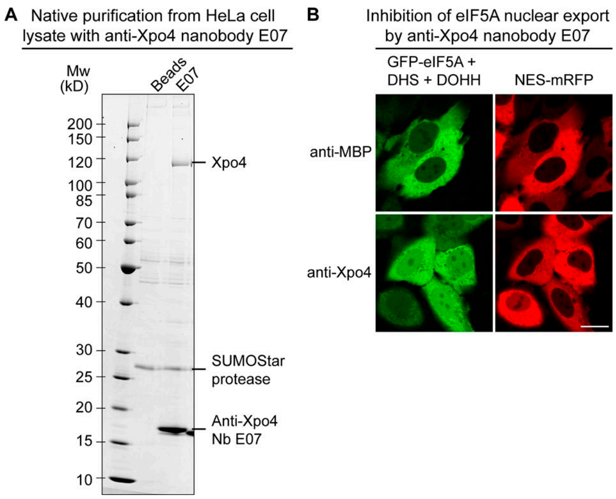

Figure S4. **Nanobody E07 recognizes Xpo4 specifically and impedes Xpo4-dependent export. (A)** Validation of the binding specificity of E07 for Xpo4 was as described for the anti-Xpo7 nanobodies (Nbs) in Fig. S1 B. **(B)** eIF5A is the main export substrate of Xpo4 (Lipowsky et al., 2000; Aksu et al., 2016). It was transiently expressed as a GFP-fusion in HeLa cells, together with the two enzymes that convert Lys^{50} of eIF5A to hypusine, namely deoxyhypusine synthase (DHS) and deoxyhypusine hydroxylase (DOHH). Hypusine is part of eIF5A's export signal. Cotransfection of E07 shifts the GFP-eIF5A localization from cytoplasmic to equilibrated. Mw, molecular weight. Bar, 20 µm.

Table S1. **Nucleus/cytoplasm GFP signal ratios of cargoes analyzed in this study**

**Categories**	**Cargoes**	**Ratios of nuclear/cytoplasmic cargo concentrations**						
		**Without Nb**	**+ MBP Nb**	**+ Xpo4 Nb**	**+ Xpo7 Nb D18**	**+ MBP Nb (2)**	**+ Xpo7 Nb D11**	**+Xpo7 D11+D18**
Xpo7 export cargo candidates	GFP-Ttc39C	0.147 ± 0.028	0.100 ± 0.024	0.123 ± 0.034	0.656 ± 0.101	0.159 ± 0.036	0.718 ± 0.108	ND
	GFP-Smyd3	0.144 ± 0.040	0.132 ± 0.052	0.129 ± 0.022	0.796 ± 0.091	0.157 ± 0.078	1.042 ± 0.147	ND
	GFP-Sufu	0.748 ± 0.170	0.554 ± 0.120	0.718 ± 0.113	1.371 ± 0.125	0.783 ± 0.071	1.495 ± 0.143	ND
	GFP-Metap1	0.169 ± 0.096	0.090 ± 0.019	0.084 ± 0.050	0.447 ± 0.093	0.140 ± 0.087	0.949 ± 0.171	ND
	GFP-Sestrin-2	0.455 ± 0.182	0.156 ± 0.034	0.209 ± 0.065	1.008 ± 0.098	0.348 ± 0.123	1.231 ± 0.103	ND
	GFP-Gpn1	ND	0.333 ± 0.104	ND	0.302 ± 0.066	ND	ND	ND
	GFP-Pex5	ND	0.153 ± 0.044	ND	0.148 ± 0.023	ND	ND	ND
Control (Xpo1 cargo)	GFP-Metap2	0.025 ± 0.011	0.051 ± 0.021	0.061 ± 0.026	0.050 ± 0.026	ND	ND	ND
Xpo7 import cargo candidates	GFP-Hat1	4.691 ± 1.140	5.828 ± 1.077	4.597 ± 0.707	0.542 ± 0.224	8.318 ± 1.980	0.530 ± 0.212	ND
	GFP-Nampt	0.715 ± 0.156	1.132 ± 0.359	0.993 ± 0.353	0.245 ± 0.041	0.995 ± 0.293	0.166 ± 0.047	ND
	GFP-Smug1	4.536 ± 1.965	6.088 ± 3.221	5.098 ± 2.783	0.773 ± 0.085	2.197 ± 0.935	0.710 ± 0.160	ND
	GFP-Hmbs	2.897 ± 1.573	5.996 ± 4.372	4.252 ± 2.940	1.115 ± 0.083	6.513 ± 3.130	1.122 ± 0.119	ND
	GFP-Hdac8	2.510 ± 0.775	3.852 ± 1.966	3.266 ± 1.314	1.314 ± 0.142	3.923 ± 1.387	1.265 ± 0.109	ND
	GFP-CutC	ND	56.317 ± 22.318	ND	11.996 ± 4.034	ND	ND	ND
	GFP-Rbbp7	ND	2.653 ± 0.970	ND	2.376 ± 0.364	ND	ND	ND
	Endogenous Hat1 (IF)	ND	6.652 ± 2.068	ND	1.658 ± 0.446	ND	1.311 ± 0.319	0.967 ± 0.205

Data are means ± SD. Nuclear and cytoplasmic GFP signals were integrated in ImageJ and used to calculate nuclear/cytoplasm GFP ratios in single cells. 4–20 cells from each sample were used to derive the listed data. IF, immunofluorescence; Nb, nanobody; NB, not determined.

Data S1 is a separate Excel file showing quantitative interaction data of ~300 potential (import and export) cargoes with Xpo7.

References

Aksu, M., S. Trakhanov, and D. Görlich. 2016. Structure of the exportin Xpo4 in complex with RanGTP and the hypusine-containing translation factor eIF5A. *Nat. Commun.* 7:11952. https://doi.org/10.1038/ncomms11952

Kırlı, K., S. Karaca, H.J. Dehne, M. Samwer, K.T. Pan, C. Lenz, H. Urlaub, and D. Görlich. 2015. A deep proteomics perspective on CRM1-mediated nuclear export and nucleocytoplasmic partitioning. *eLife.* 4:e11466. https://doi.org/10.7554/eLife.11466

Lipowsky, G., F.R. Bischoff, P. Schwarzmaier, R. Kraft, S. Kostka, E. Hartmann, U. Kutay, and D. Görlich. 2000. Exportin 4: a mediator of a novel nuclear export pathway in higher eukaryotes. *EMBO J.* 19:4362–4371. https://doi.org/10.1093/emboj/19.16.4362

Pleiner, T., M. Bates, and D. Görlich. 2018. A toolbox of anti-mouse and anti-rabbit IgG secondary nanobodies. *J. Cell Biol.* 217:1143–1154. https://doi.org/10.1083/jcb.201709115
